# An Explainable Machine Learning Pipeline for Stroke Prediction on Imbalanced Data

**DOI:** 10.3390/diagnostics12102392

**Published:** 2022-10-01

**Authors:** Christos Kokkotis, Georgios Giarmatzis, Erasmia Giannakou, Serafeim Moustakidis, Themistoklis Tsatalas, Dimitrios Tsiptsios, Konstantinos Vadikolias, Nikolaos Aggelousis

**Affiliations:** 1 Department of Physical Education and Sport Science, Democritus University of Thrace, 69100 Komotini, Greece; 2 AIDEAS OÜ, Narva mnt 5, 10117 Tallinn, Estonia; 3 Department of Physical Education and Sport Science, University of Thessaly, 38221 Trikala, Greece; 4 Department of Neurology, School of Medicine, University Hospital of Alexandroupolis, Democritus University of Thrace, 68100 Alexandroupolis, Greece

**Keywords:** stroke, clinical data, machine learning, prognosis, interpretation

## Abstract

Stroke is an acute neurological dysfunction attributed to a focal injury of the central nervous system due to reduced blood flow to the brain. Nowadays, stroke is a global threat associated with premature death and huge economic consequences. Hence, there is an urgency to model the effect of several risk factors on stroke occurrence, and artificial intelligence (AI) seems to be the appropriate tool. In the present study, we aimed to (i) develop reliable machine learning (ML) prediction models for stroke disease; (ii) cope with a typical severe class imbalance problem, which is posed due to the stroke patients’ class being significantly smaller than the healthy class; and (iii) interpret the model output for understanding the decision-making mechanism. The effectiveness of the proposed ML approach was investigated in a comparative analysis with six well-known classifiers with respect to metrics that are related to both generalization capability and prediction accuracy. The best overall false-negative rate was achieved by the Multi-Layer Perceptron (MLP) classifier (18.60%). Shapley Additive Explanations (SHAP) were employed to investigate the impact of the risk factors on the prediction output. The proposed AI method could lead to the creation of advanced and effective risk stratification strategies for each stroke patient, which would allow for timely diagnosis and the right treatments.

## 1. Introduction

One of the most common causes of early death, stroke can be organized into two main categories: (i) ischemic stroke and (ii) hemorrhagic stroke [1]. In general, fatalities in stroke patients are observed in up to 23% of cases [2]. Despite the fact that stroke is highly correlated with age, stroke mortality rates for men and women are comparable below the age of 45 years, in contrast with the higher risk of stroke for men between 45 and 74 years [3]. In a recent study, Khan et al. found that COVID-19 infection in children is a new risk factor that could lead to the occurrence of an ischemic stroke [4]. Furthermore, stroke is the second largest cause of secondary disabilities, including impaired speech, cognitive problems, and loss of mobility [5]. These kinds of disabilities lead to reduced quality of life. In particular, the human functions that are most affected by stroke are those related to motor skills. The recovery phase lasts more than 6 months, and it should be stressed out that only a small percentage of survivors (up to 20%) will achieve full functionality of the affected upper limbs and 83% of them will be able to walk again [6]. From a different perspective, Strilciuc et al. also found that the economic burden of stroke is significant [7]. Specifically, in Europe, the cost of productivity loss following stroke amounted to EUR 12 billion and the cost of health care was estimated at EUR 27 billion for 2017.

Stroke affects both men and women, reducing their quality of life and burdening the public health system. Due to the high impact on society, the scientific community emphasizes the development of models for predicting strokes with the aim of preventing them. In this regard, AI plays a key role since its use is now widespread in the prevention of various diseases [8,9,10]. According to a recent literature review, several studies have been carried out to develop models for diagnosing stroke [11,12,13] or predicting treatment responses and patient outcomes, with the ultimate objective of forming personalized rehabilitation protocols [14,15,16]. Arslan et al. proposed a data mining approach for ischemic stroke prediction which is based on 80 subjects with ischemic stroke and 112 healthy subjects [17]. The best performance (97.89% accuracy and 97.83% AUC) was achieved by the Support Vector Machine (SVM) classifier, and an analysis of feature importance was also conducted to identify risk factors that are mainly associated with ischemic stroke.

In another study, Liu et al. worked on imbalanced data and proposed a hybrid ML approach for the prediction of cerebral stroke [18]. They used physiological data (783 stroke patients from a dataset of 43.400 subjects) to train a deep neural network (DNN) optimized via an automated hyperparameter tool (AutoHPO). They achieved an accuracy of 71.6% and a false-negative rate of 19.1%. Zhao et al. proposed a DNN approach to predict the risk of pre-operative acute ischemic stroke. Using a combination of clinical data, transthoracic echocardiography, and CTA imaging, they achieved a 96.4% AUC score [19]. Furthermore, Alanazi et al. worked on the task of predicting the risk of stroke on an imbalanced clinical dataset (biomarkers) from the National Health and Nutrition Examination Survey (NHANES). Four ML classifiers were tested, and the optimal accuracy (96%) was finally achieved by the Random Forest (RF) algorithm [20]. Moreover, Cui et al. proposed an ML-based model for predicting the incidence and severity of acute ischemic stroke in patients with anterior circulation large vessel occlusion [21]. They explored the effectiveness of four well-known classifiers on an imbalanced clinical dataset, achieving ROC-AUC scores of up to 67% on an external dataset.

Despite their widespread use, machine learning (ML) and deep learning (DL) models are characterized as black boxes, with only a few studies in medical applications focusing on the interpretation of the ML models’ output [22,23,24]. Specifically, Kim et al. proposed an interpretable ML model for predicting early neurological deterioration in atrial fibrillation (AF)-related strokes [25]. They used clinical data from 2363 patients with early neurological deterioration (END) and achieved a 77.2% score for the area under the receiver operating characteristic curve (AUROC). Applying SHAP, they demonstrated that the most influential factors were the National Institute of Health Stroke Scale score and the fasting glucose level. So far, a lack of transparency has been identified as a major implementation barrier, preventing clinicians from accepting AI-generated decisions or recommendations. In order to fill the research gap, we propose an explainable machine learning pipeline for stroke prediction based on an extremely imbalanced dataset with routine clinical measurements. The proposed methodology relies on SHAP, a popular explainable AI (XAI) framework offering model-agnostic explanations. It is based on Shapley values, a notion frequently used in cooperative game theory. Our XAI technique quantifies the importance of each feature as the average marginal contribution over all feasible coalitions. Overall, we propose an end-to-end XAI methodology that comprises of the following steps: (i) feature engineering to standardize data and remove noise, (ii) learning on a comparative analysis that involves several well-known ML models, and (ii) validation and explainability to evaluate the predictive performance of the trained ML models and quantify the contribution of each input feature to stroke prediction. Apart from the prediction task, secondary objectives of the current study are (i) to apply data resampling techniques to cope with an extremely imbalanced dataset and (ii) to reduce the false-negative rate in the prediction of stroke. The rest of the paper is organized as follows. Materials and methods are described in Section 2. Section 3 demonstrates the results of the proposed explainable ML pipeline. The results are presented in Section 4, and the conclusions are drawn in Section 5.

## 2. Materials and Methods

### 2.1. Data Description

Data were obtained from Kaggle’s public dataset “Cerebral Stroke Prediction-Imbalanced Dataset” (https://www.kaggle.com/shashwatwork/cerebral-stroke-prediction-imbalaced-dataset, accessed on 1 December 2021) [18]. The dataset contains a total of 43,400 subjects and 10 risk factors for stroke incidence, including the target variable “stroke”. The detailed dataset description is presented in Table 1.

### 2.2. Problem Definition

**Figure 1:**
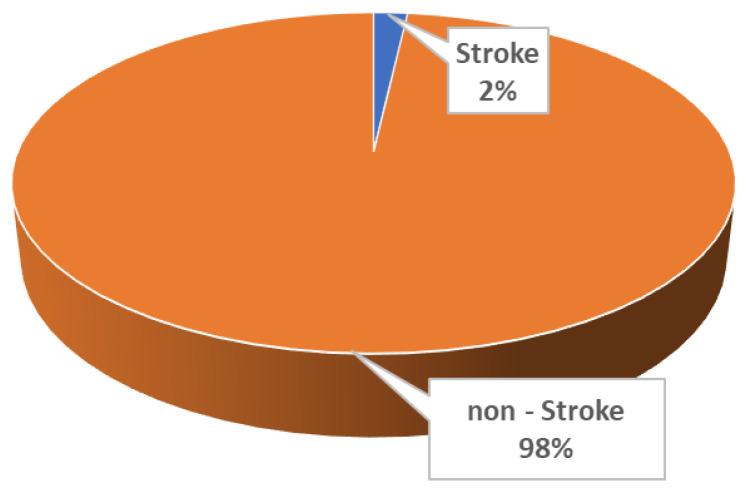
Rates of classes in the binary problem.

In this study, we focused on the development of an explainable machine learning pipeline that could identify important risk factors that contribute to the prediction of stroke and their impact on model output with a focus on post hoc explainability. For this aim, we consider the stroke prediction task as a two-class classification problem. Specifically, the subjects of this study were divided into two classes: (i) the stroke occurrence class, consisting of subjects with the occurrence of stroke, and (ii) the non-stroke class, which includes healthy subjects without confirmed stroke (Figure 1).

### 2.3. Proposed Methodology

**Figure 2:**
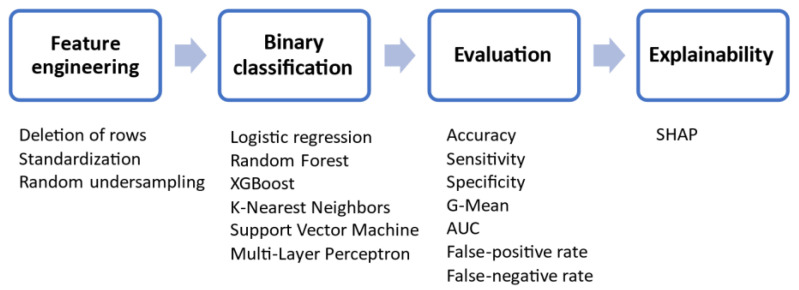
Workflow of the proposed AI methodology.

The proposed AI methodology for the prediction and interpretation of stroke occurrence includes four processing steps: feature engineering of the collected clinical data (10 risk factors in total), learning process, evaluation of the classification results, and explainability/interpretation analysis. The workflow of the proposed methodology is presented in Figure 2. The codes for the preprocessing of the data, the implementation of the ML models, and the explainability analysis were implemented in Python 3.6 by using scikit-learn 0.24.2 (https://scikit-learn.org/0.24/, accessed on 15 December 2021).

### 2.4. Feature Engineering

To be consistent with the study of Liu et al. [18], we first removed outliers’ values from the risk factors “age” (10 years) and “BMI” (>60). Then, in order to cope with the missing values and given the massive availability of data in the current study (43,400 samples), we deleted all records that contained missing values [26]. Furthermore, as a standardization technique, we employed the StandardScaler function from scikit-learn 1.0.2 [27]. This technique standardizes the risk factors by removing the mean and scaling them to unit variance.

### 2.5. Learning

For the prediction task, six well-known ML classifiers which are widely used in medical applications were employed: Logistic Regression (LR) [28], Random Forest (RF) [29], XGBoost [30], K-Nearest Neighbors (KNN) [31], Support Vector Machine (SVM) [32], and Multi-Layer Perceptron (MLP) [33].

### 2.6. Validation and Evaluation Metrics

To evaluate the prediction performance of the proposed ML classifiers, a nested stratified 10-fold cross-validation process was adopted [34]. In order to form balanced binary datasets for training, random under-sampling was applied to the majority class in each of the ten training data folds. The one remaining testing fold remained intact. Model fitting and hyperparameter optimization were applied to the training data folds using the GridSearchCV function from scikit-learn 1.0.2. In Table 2, the employed hyperparameters for each ML model are presented. The proposed learning approach (under-sampling, hyperparameter optimization on training, and validation on the testing fold) was applied ten times (one per fold) and the validation results were combined over the rounds, providing a performance on the whole imbalanced dataset.

Prediction accuracy, sensitivity, specificity, G-Mean, and AUC were employed for objective evaluation of the competing classifiers [35]. The false-positive rate (FP_rate_) and the false-negative rate (FN_rate_) were also considered in our study given their important role in medical applications [36]. False-positive decisions may lead to expensive follow-up testing or even unnecessary medical treatment. On the other hand, false negatives put patients at risk of not receiving appropriate treatment on time since their disease remains undiagnosed. Given the extremely imbalanced dataset of our study, it is expected that some of the aforementioned metrics might reveal more about the class distribution than the actual performance of the trained models. In light of this, we considered a combination of metrics to select the optimal ML model, giving priority to (i) G-Mean, which balances the performances between the majority and minority classes, and (ii) FN_rate_, which classifies patients as healthy while they may be in danger, and they may not receive appropriate treatment on time since their disease remains undetected.

### 2.7. Explainability

As a final step of the proposed methodology, we employed the Shapley Additive Explanations (SHAP) model to perform post hoc explainability analysis on the best performing ML model. SHAP is an explanation tool based on game theory for the output of any ML model [37,38,39]. It provides a link between optimal credit distribution and local explanations by employing basic Shapley values from game theory. SHAP aims to explain the prediction of an instance by estimating the contribution of each input variable with Shapley values that are computed using coalitional game theory. Instead of building a model that predicts using many inputs, the problem should be seen as a game in which each feature (“player”) contributes to the prediction process (“score”). SHAP estimates the number of points we would earn or lose in the presence or absence of a feature to identify the contribution of each player (feature) in predicting the score. To determine the Shapley value of a particular feature, a weighted sum of the differences in scores between games (predictions) in which the player (feature) plays and games from which the player (feature) is removed is calculated.

In this study, we evaluated how risk factors affect the final stroke prediction. To this end, we employed SHAP to score features based on their influence on the optimum ML outputs and to build a small explanatory model, which adds to our understanding of the relative impact of features on stroke prediction.

## 3. Results

In this section, we summarize the prediction performance results of the proposed ML models. Furthermore, the impact of the risk factors on the classification result of the best performing model is discussed by employing the SHAP model.

### 3.1. Comparative Analysis

Table 3 summarizes the prediction performance results of the comparative analysis of the six well-known classifiers. The LR classifier achieved the best scores in accuracy (73.52%), specificity (73.43%), AUC (83.30%), and FP_rate_ (26.57%). The MLP classifier, on the other hand, had the highest sensitivity (81.40), G-Mean (75.83%), and FN_rate_ (18.60%) scores. Moderate scores were obtained by the RF, XGBoost, and SVM classifiers. Finally, the KNN classifier recorded the lowest performance in the majority of metrics (accuracy of 69.16% and G-Mean of 73.70%).

**Figure 3:**
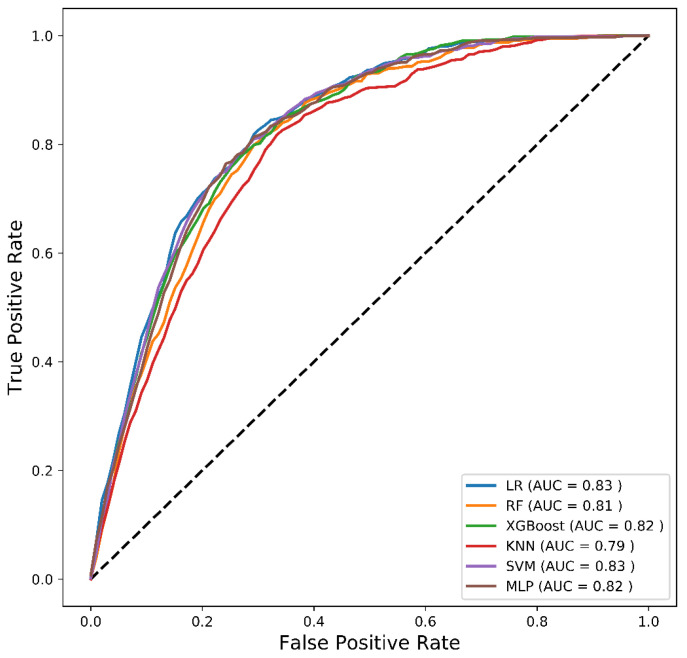
Mean ROC curves of the employed classifiers.

**Figure 4:**
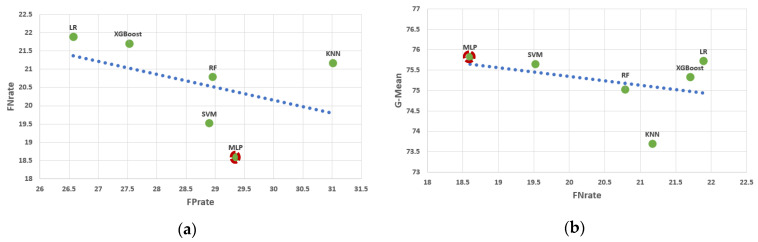
(**a**) Scatter plot of FP_rate_ and FN_rate_. (**b**) Scatter plot of FN_rate_ and G-Mean.

Figure 3 depicts the mean ROC curves of the employed classifiers in this binary problem. We can observe that AUC scores ranging from 79% to 83% were achieved by the employed models. From a different perspective, Figure 4 demonstrates the relationship between FP_rate_ and FN_rate_ in (a) and between FN_rate_ and G-Mean in (b). The following conclusions can be drawn from Figure 4. (i) We observe an almost inverse relationship between the FN_rate_ and the FP_rate_. Specifically, as the FN_rate_ decreases, the FP_rate_ increases and vice versa, with the exception of KNN. (ii) MLP achieved the lowest FN_rate_ and the second highest FP_rate_. (iii) LR was the best performer with respect to FP, but simultaneously the worst performer in FN_rate_. (iv) MLP had the highest G-Mean while also having the lowest FN_rate_. Thus, the MLP classifier was chosen as the best ML model for this binary problem because it found the best balance between the two most important metrics (G-Mean and FN).

### 3.2. Explainability Results

**Figure 5:**
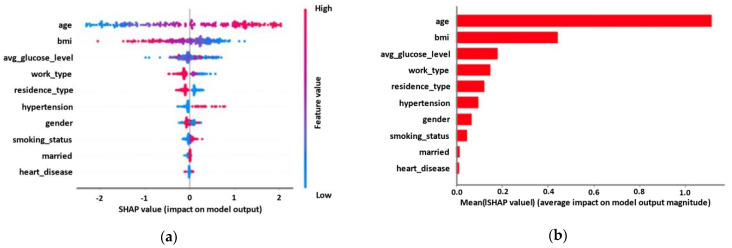
Risk factors’ impact on MLP model output for the cerebral stroke prediction dataset. (**a**) The distribution of the impact of a risk factor value on the model output across test instances. (**b**) The average impact magnitude for test instances.

**Figure 6:**
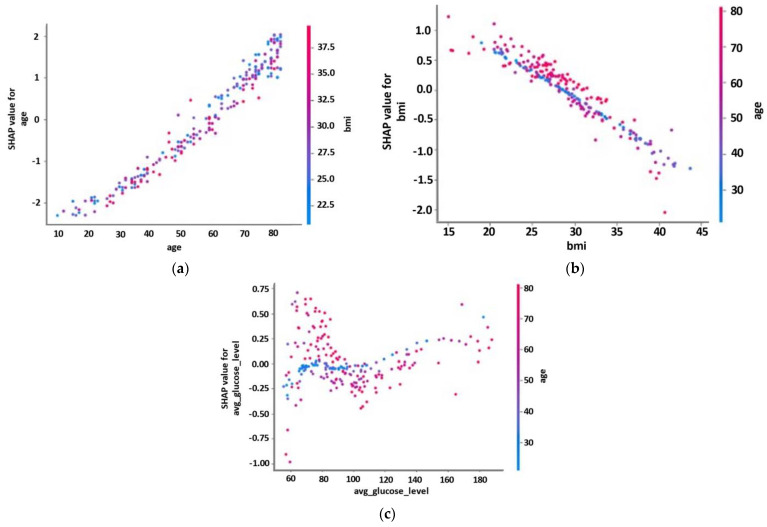
SHAP dependence plots of three representative risk factors: (**a**) age; (**b**) BMI; (**c**) avg_glucose_level.

Figure 5 depicts the risk factors’ impact on the best performing model’s (MLP) output. Figure 6 depicts the negative and positive relationships of the risk factors with the target (occurrence of stroke). A positive relationship is actually defined when the increase in a feature value is correlated with an increase in the model’s output and vice versa for negative relationships. Moreover, the SHAP summary values of the risk factors in Figure 5a are shown in descending order in a top–down view, with the most impactful risk factors at the top. The color represents whether the value of the risk factor is high (red) or low (blue) for each observation individually. The risk factor age shows that the patients’ age has a high and positive impact on the occurrence of stroke (e.g., as age increases, this pushes the model output to increase towards the class of patients with stroke), so an increase in age is associated with the occurrence of stroke. On the other hand, BMI is negatively correlated with the occurrence of stroke. Figure 5b depicts the average impact of each risk factor on the model’s output magnitude. As it is observed, age, BMI, and avg_glucose_level have major contributions to the model’s output, whereas factors such as work_type, residence_type, hypertension, and gender have moderate to low impact on the model’s output.

Partial dependence plots were also generated (Figure 6) to quantify the marginal effect of the three most important risk factors on the predicted output of the MLP model. These plots demonstrate the kind of relationship between the occurrence of a stroke and the risk factors (e.g., monotonic, linear, or more complex). Figure 6a depicts a linear and positive relationship between age and the occurrence of stroke. A linear but negative relationship was observed for BMI with the occurrence of stroke. The average glucose level had a minor effect, with the obtained SHAP values within the range of [−1, 0.75], whereas the association with the incidence of stroke revealed a more complicated non-linear relationship.

## 4. Discussion

This work focuses on the development of an explainable ML pipeline for the prediction of stroke occurrence. This prediction task was tackled as a binary classification problem where the subjects of the employed dataset were divided into two classes (stroke and non-stroke). In order to perform the binary classification task (stroke versus non-stroke), various ML models were employed, and we achieved a low false-negative rate of 18.6% and a satisfactory G-Mean score (75.83%). Another major objective of this study was to identify informative risk factors that significantly contribute to the classification output (stroke prediction).

To cope with the imbalanced dataset, we applied a well-established data resampling technique (e.g., random under-sampling in each individual run) in combination with a nested stratified 10-fold cross-validation process. To evaluate the predictive performance of the proposed approach, six ML models were employed. The best accuracy (73.52%), specificity (73.43), and AUC (83.30) scores were achieved by the LR model, whereas the MLP model recorded the best sensitivity and G-Mean scores, 81.4% and 75.83%, respectively (Table 3). Despite the fact that the LR model is a computationally efficient classifier, we selected the MLP classifier as the best ML model for this medical task because this model achieved the best trade-off between FN_rate_ and G-Mean in the comparative analysis (Figure 4). Our approach achieved overall better scores in comparison to the study of Liu et al., where the main assessment scores were the FN_rate_ and the FP_rate_. Specifically, on the same dataset, we achieved 18.6% instead of 19.1% and 31.02% instead of 33.1% for FN_rate_ and FP_rate_, respectively [18].

During this study, the risk factors that mainly shaped the predictive ability of our best ML model were also identified. The SHAP model found that age, BMI, and the average glucose level were the three most important predictors of risk. The rest of the risk factors had low or minor contributions (Figure 5). The majority of the existing studies include age as one of the most important risk factors in the occurrence of stroke. Yousufuddin et al. stated that aging is the most robust non-modifiable risk factor for incident stroke [40]. BMI was also identified as the second most important risk factor. Despite the fact that obesity is highly associated with an increased risk of stroke incidents, recent findings in the literature show that leaner adults (lower BMI) have higher mortality rates than overweight or obese adults [41]. In another study, Park et al. also demonstrated that the risk of any type of stroke decreased in obese patients [42]. Furthermore, the average glucose level was selected as a high-impact risk factor. According to Zheng et al., the average glucose level is strongly associated with the occurrence of an ischemic stroke [43].

Work type, residence, hypertension, gender, and smoking status display lower impact than the aforementioned risk factors in our model. Hypertension or high blood pressure is highly correlated with the occurrence of stroke (e.g., ischemic stroke) [44]. The type of work can also be characterized as a risk factor. Huang et al. stated that high-strain jobs are correlated with an increased risk of stroke [45]. In addition, despite the fact that residence has a low impact as a risk factor in stroke in our study, Sealy-Jefferson et al. concluded that postmenopausal women who lived in rural areas had a higher stroke risk [46]. Gender presents differences in stroke rates among women and men [3]. According to Peters et al., men continue to have a higher stroke incidence rate than women [47]. In another study, Shah et al. demonstrated that smoking status increases the risk of stroke [48]. Overall, the low but not negligible contribution of risk factors resulting from the present study is also confirmed by the existing literature.

As a limitation, the implementation of a nested cross-validation strategy is costly in computational terms. However, this is an offline process that needs to be performed once, and the inference time of the trained model will be small, enabling the almost real-time application of the ML models. The whole feature set was utilized in the proposed analysis, and this could also be seen as a limitation. We did not employ any feature selection because we wanted to quantify the contribution (SHAP values) of each feature to the stroke prediction outcome. This information is valuable for the design of an optimized future experimental setup that would be based on the most relevant risk factors. Furthermore, the lack of an external validation dataset for evaluating the generalization of the best ML model could be characterized as a limitation. Our future work includes the development of a database with easy-to-read and low-cost measurements and potentially complementary data (such as genetic factors), which will be based on a stratified sampling of the general population in Greece. Utilizing the new database, we will identify subpopulations of participants who are at higher risk of having a stroke (either ischemic or hemorrhagic stroke). This dataset could also serve as a validation set to further evaluate the predictive accuracy of the proposed ML models on unseen (unknown) real-world data. In order to achieve these goals, it is necessary to employ more advanced AI tools (e.g., pre-trained CCN or Siamese neural networks), feature selection techniques, and interpretation approaches using graphical algorithms.

## 5. Conclusions

In this study, we proposed a reliable ML prediction model for stroke disease, and we coped with the challenge of following and tracing the logic of the ML algorithms. In a severely imbalanced dataset, we achieved a competitive FN_rate_ score (18.6%) in comparison with the existing studies. The proposed explainable ML methodology was based on a well-established ML pipeline that combines data resampling techniques to cope with the observed class imbalance data problem with explainability analysis via SHAP. Understanding the mechanisms behind stroke occurrence via advanced ML techniques is a valuable tool for creating more reliable, non-invasive, and powerful prediction tools in the hands of clinicians.

## Figures and Tables

**Table 1:** Included risk factors.

Risk Factors	Description	Type of Variable
Age	Current age	Continuous
BMI	Body mass index (BMI) is a measure of body fat	Continuous
Gender	Female/Male	Categorical
Average glucose	Average glucose is an estimated average of blood sugar	Continuous
Work type	Never worked/Children/Government job/Self-Employed/Private	Categorical
Residence type	Rural/Urban	Categorical
Smoking status	Never/Formerly/Smoker	Categorical
Heart disease	No/Yes	Categorical
Married	No/Yes	Categorical
Hypertension	No/Yes	Categorical

**Table 2:** ML hyperparameters tested in our experimentation.

ML Model	Description
LR	C: [0.01, 0.1, 1, 10, 100], penalty: [‘l1’, ‘l2’]
RF	criterion: [‘gini’, ‘entropy’], min_samples_leaf: [1, 2, 3, 4, 5], min_samples_split: [2, 3, 4, 5, 6, 7], n_estimators: [10, 15, 20, 25, 27, 30]
XGBoost	max_depth: [1, 2, 3, 4, 5, 6, 7, 8, 9, 10], min_child_weight: [1, 2, 3, 4, 5, 6, 8, 10], gamma: [0, 0.4, 0.5, 0.6,0.7,0.8,0.9,1]
KNN	algorithm: [‘auto’, ‘ball_tree’, ‘kd_tree’, ‘brute’], leaf_size: [1, 2, 3, 5], n_neighbors: [3, 4, 5, 7, 9, 12, 14, 15, 16, 17], weights: [‘uniform’, ‘distance’]
SVM	kernel: [‘rbf’, ‘linear’, ‘sigmoid’], C: [0.001, 0.1, 0.1, 10, 25, 50, 100, 1000], gamma: [0.01, 0.001, 0.0001, 1 × 10^−5^]
MLP	activation: [‘tanh’, ‘relu’], alpha: [0.0001, 0.05], hidden_layer_sizes: [(2, 5, 10), (5, 10, 20), (10, 20, 50)], learning_rate: [‘constant’, ‘adaptive’], solver: [‘sgd’, ‘adam’]

**Table 3:** Mean results of prediction models (%).

ML Models	Accuracy	Sensitivity	Specificity	G-Mean	AUC	FP_rate_	FN_rate_
LR	**73.52**	78.12	**73.43**	75.73	**83.30**	**26.57**	21.89
RF	71.19	79.22	71.04	75.02	81.24	28.96	20.79
XGBoost	72.58	78.30	72.47	75.33	82.50	27.53	21.70
KNN	69.16	78.84	68.98	73.70	79.35	31.02	21.17
SVM	71.28	80.48	71.10	75.65	82.85	28.90	19.52
MLP	70.85	**81.40**	70.65	**75.83**	82.14	29.35	**18.60**

## Data Availability

Publicity data from the Kaggle are available at https://www.kaggle.com/shashwatwork/cerebral-stroke-predictionimbalaced-dataset (accessed on 1 December 2021).
